# Blood compatibility evaluation of polydopamine nanoparticles

**DOI:** 10.3389/fphar.2025.1530650

**Published:** 2025-02-28

**Authors:** Sha Li, Si-Ming Yan, Li-Wei Zhang, Xiao-Yan Yang, Zhong Guo

**Affiliations:** ^1^ Zhuhai Key Laboratory of Basic and Applied Research in Chinese Medicine, School of Bioengineering, Zunyi Medical University, Zhuhai, Guangdong, China; ^2^ Center for Biological Science and Technology, Faculty of Arts and Sciences, Beijing Normal University, Zhuhai, Guangdong, China

**Keywords:** polydopamine nanoparticles, blood compatibility, concentration-dependent effect, acute toxicity, intravenous injection

## Abstract

**Introduction:**

Polydopamine nanoparticles (PDA NPs) exhibit numerous outstanding characteristics, including simple preparation, broad light absorption, drug binding ability, excellent biocompatibility and adhesive properties, making them suitable for biomedical application. However, the limited information on their hemocompatibility may hinder their progression from laboratory research to clinical application.

**Methods:**

In this study, we investigated comprehensively the hemocompatibility of PDA NPs, assessed the effects of PDA NPs on red blood cells (RBCs) morphology and lysis, fibrinogen structure and conformation, blood coagulation, platelet activation, complement system activation, and organ toxicity.

**Results:**

The results indicated that PDA NPs can induce morphological changes and hemolysis in RBCs in a concentration-dependent manner. Interactions with fibrinogen suggested a disturbance in the protein’s microenvironment without significantly altering its secondary structure. This study also revealed that PDA NPs have a concentration-dependent effect on blood coagulation, platelet activation, and complement system activation. Additionally, PDA NPs showed no significant acute toxicity after intravenous injection.

**Conclusion:**

The findings offer important insights into the hemocompatibility of PDA NPs, which is essential for their safe and effective clinical use. Understanding their interactions with blood components is key to ensuring their compatibility in biomedical applications. These results are vital for guiding the development of PDA NPs for medical use, particularly in blood-contacting applications.

## Graphical Abstract

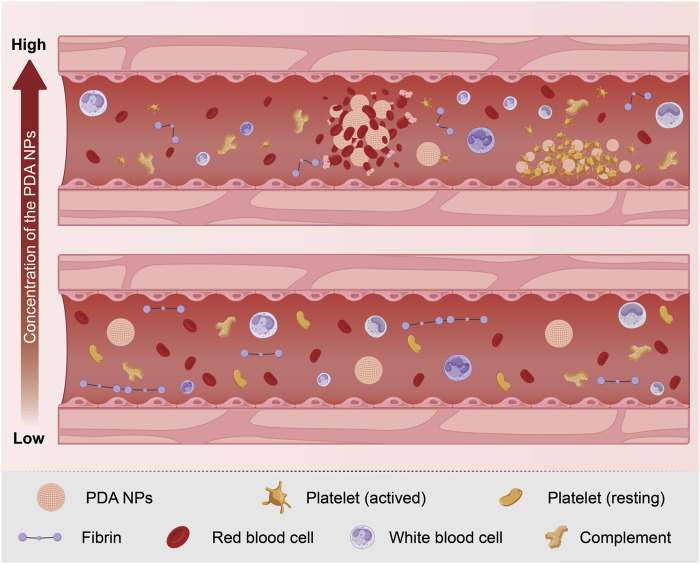

## 1 Introduction

The advancement of nanotechnology and biomaterials has greatly propelled biomedical applications, particularly in drug delivery, tissue engineering, and medical device manufacturing (Han et al., 2022). Biomaterials, due to their unique size, shape, and surface characteristics, are increasingly being integrated into therapeutic systems and diagnostics. Among various synthetic biomaterials, polydopamine nanoparticles (PDA NPs) have emerged as promising candidates in biomedical application due to their remarkable physicochemical properties and ease of synthesis (Xu et al., 2023). PDA NPs, derived from dopamine through a simple oxidative polymerization process under mildly alkaline conditions, are melanin-like, mussel-inspired biomaterials that possess a range of beneficial features (Cheng et al., 2019). They exhibit several advantageous properties, such as easy preparation, low toxicity, strong adhesion to various substrates, excellent photothermal conversion efficiency, versatile surface chemistry due to abundant functional groups (including catechol and amino groups), and potent free radical scavenging activities, especially against reactive oxygen species (ROS) (Liu Y. et al., 2013; El Yakhlifi and Ball, 2020; Hu et al., 2020). These properties have facilitated significant research developments in the fields of drug delivery, implant surface modification, cancer therapy, theranostics, antibacterial treatments, and autoimmune disease management, demonstrating the wide range of biomedical applications for which PDA NPs are suitable (Huang et al., 2018; Jia et al., 2019; Yang et al., 2015; Wu et al., 2023; Kwon and Bettinger, 2018; Djermane et al., 2022).

Biocompatibility is a critical requirement for the application of biomaterials in both *in vitro* and *in vivo* biomedical settings. Owing to their structural similarity to natural melanin, PDA NPs demonstrate favorable biocompatibility, showing non-toxicity to cells and tissues, minimal immune response when introduced into the body, and the potential for gradual degradation over time (Wu et al., 2023; Liu et al., 2020; Liu X. et al., 2013; Lin et al., 2021; Hauser et al., 2020). The large number of catechol and amino groups in PDA NPs enhances their ability to adhere to almost all types of organic and inorganic materials, thus frequently being used to modify surfaces to improve biocompatibility and reduce systemic toxicity (Wu et al., 2018; Mengdi et al., 2022). While PDA NPs display good biocompatibility, particularly in cellular interactions (Liang et al., 2023), their hemocompatibility poses certain limitations, especially for blood-contacting applications. Despite their antifouling properties, PDA NPs may permit some degree of platelet activation and adhesion upon contact with blood (Luo et al., 2013), potentially leading to thrombus formation and increasing the risk of thrombosis and related complications (Lynch and Dawson, 2008; Zhong et al., 2013). Additionally, PDA NPs have the potential to interact with various blood components, including plasma proteins, RBCs, platelets, and the complement system. These interactions may lead to changes in the functionality of these blood components, triggering inflammatory responses or altering blood coagulation processes (Lynch and Dawson, 2008). Therefore, a comprehensive evaluation of the hemocompatibility of PDA NPs is necessary to ensure their safety, efficacy, and clinical viability.

This study synthesizes and characterizes PDA NPs, investigating their effects on the structure and function of key blood components, including RBC morphology and lysis, fibrinogen structure and conformation, blood coagulation, platelet activation, complement system activation, and acute toxicity. These findings aim to provide valuable insights into the blood compatibility of PDA NPs, guiding their design and clinical application.

## 2 Materials and methods

### 2.1 Materials

Ethanol absolute and inulin were purchased from Aladdin (Shanghai, China). Ammonia water was provided by Tianjin Damao Chemical Reagent Factory (Tianjin, China). Dopamine, fibrinogen and polyethyleneimine (Mw 600) were purchased from Sigma-Aldrich (MO, United States). Fresh whole blood from healthy consented volunteers was collected in sodium citrate tubes. Healthy C57BL/6 mice (4–6 weeks old) were provided by Beijing Huafukang biotechnology company (Beijing, China). All animal experiments in this study were approved by the Committee on the Use of Live Animals in Teaching and Research of Zunyi University and conformed to institutional and governmental guidelines and regulations.

### 2.2 Preparation and characterization of PDA NPs

An ethanol solution (50%, v/v) was prepared, and its pH was adjusted to 8.5 using ammonia water. Then, dopamine (4 mg/mL) was dissolved in this ethanol solution and stirred at 600 rpm for 2 h to prepare PDA NPs. After stirring, the reaction mixture was centrifuged at 10,000 rpm for 15 min and washed twice with deionized water. The black precipitate was collected and lyophilized to obtain PDA NPs.

The morphology of PDA NPs was observed by using a scanning electron microscope (SEM) (Hitachi, Regulus 8100, Japan). The zeta potential and hydration particle size of PDA NPs in deionized water were measured with a zeta potential analyzer (Malvern, Zetasizer Nano ZS, United Kingdom). The chemical structures and surface functional groups of PDA NPs were characterized by using a Fourier Transform Infrared Spectrometer (FTIR) (Bruke, VERTEX 70, Germany) with the potassium bromide (KBr) pellet technique.

### 2.3 Effect of PDA NPs on RBCs morphology and lysis

#### 2.3.1 Effect of PDA NPs on RBCs morphology

Fresh anti-coagulated whole blood was collected, and centrifuged (1,000 g, 5 min) to obtain red blood cells (RBCs) pellet. The RBCs pellet was washed three time with phosphate buffered saline (PBS, pH 7.2–7.4), and incubated with either PBS (negative control) or different concentrations of PDA NPs solutions (in PBS) for 10 min at room temperature. After incubation, the RBCs/PDA NPs mixture was washed and centrifuged again to collect the RBCs. The collected RBCs were then fixed with 4% formaldehyde overnight at 4°C, and progressively dehydrated with ethanol solutions of different volume fractions (75, 85, 95, 100%). Finally, the RBCs morphology was observed using a SEM (Zeiss, Gemini300, Germany).

#### 2.3.2 Effect of PDA NPs on RBCs lysis

A 16% (v/v) RBCs suspension in PBS was pre-prepared for the RBCs lysis experiment. Briefly, 1 mL of various concentrations of PDA NPs solutions (in PBS) were incubated with 50 μL of the RBCs suspension at 37°C for a specified duration. The PBS or deionized water treatment group was considered as a negative control or positive control (complete hemolysis), respectively. Different concentrations of PDA NPs groups (without RBCs) were set as blank groups for background deduction. After incubation, the supernatants (200 μL per sample) were obtained by centrifugation (1,000 g, 5 min), and measured the absorbance values at 540 nm (A_540 nm_) using a microplate reader. The hemolysis was determined by comparing the A_540 nm_ of tested group with that of positive control.

### 2.4 Effect of PDA NPs on fibrinogen structure and conformation

#### 2.4.1 Ultraviolet (UV) absorption spectroscopy and fluorescence spectroscopy

A fibrinogen protein solution (1 mg/mL) was mixed with an equal volume of PDA NPs solutions at different concentrations (0, 0.01, 0.1, 0.2, 0.5 mg/mL), respectively. Corresponding concentrations of PDA NPs solution in PBS were used as blank controls to account for the background. The UV-vis absorption spectra of fibrinogen were measured using a UV-visible spectrophotometer (Shimadzu, UV-2550, Japan). The fluorescence emission spectra of fibrinogen were recorded using a fluorescence spectrophotometer (Hitachi, F-7000, Japan) with measurement parameters: voltage of 500 V, scan rate of 1,200 nm/min, slit width of 5 nm, λ_exc_ of 280 nm, and λ_em_ range from 300 to 650 nm.

#### 2.4.2 Circular dichroism (CD) spectroscopy

A solution of fibrinogen protein (0.4 mg/mL) was mixed with an equal volume of PDA NPs at different concentrations (0, 0.05, 0.01, 0.1, 0.2, 0.5 mg/mL), respectively. Corresponding concentrations of PDA NPs solution in PBS was set as a blank group for deducting the background. The CD spectra of fibrinogen were obtained using a CD spectrometer (Applied Photophysics, Chirascan™ V100, UK) at 25°C in a nitrogen atmosphere, scanning from 190 to 260 nm at a scan rate of 60 nm/min.

### 2.5 Effect of PDA NPs on blood coagulation

#### 2.5.1 Activated partial thromboplastin time (APTT), prothrombin time (PT) and thrombin time (TT) assays

Fresh whole blood was centrifuged at 3,000 g for 10 min to obtained platelet-poor plasma (PPP). The prepared PPP (270 μL) was incubated with various concentrations of PDA NPs solution (30 μL in PBS) at 37°C. Corresponding detection reagents were then added to the samples. Subsequently, the APTT, PT and TT of the samples were measured using an automatic coagulation analyzer (Stago, STA-R evolution, France).

#### 2.5.2 Thromboelastography (TEG) assay

Fresh citrate whole blood (900 μL) was mixed with various concentrations of PDA NPs solutions (100 μL) in kaolin-containing tubes. Subsequently, 340 μL of the PDA NPs/blood mixture and 20 μL of 0.2 M CaCl_2_ solution were added to a TEG cup. The dynamic coagulation process of the blood at 37°C in the presence of PDA NPs was recorded using a thrombelastograph coagulation analyzer (Haemoscope, TEG-5000, United States).

### 2.6 Activation of platelets by PDA NPs

Fresh whole blood was centrifuged at 100 g for 5 min to obtained platelet-rich plasma (PRP). The prepared PRP was diluted with PBS at a 1:1 volume ratio. The diluted PRP (90 μL) was co-incubated with various concentrations of PDA NPs solution (0, 0.1, 1, 10 mg/mL, 10 μL in PBS) at 37°C for 30 min. After incubation, 10 μL of the treated PRP was diluted with 90 μL of N-(2-hydroxyethyl) piperazine-N′-(2-ethanesulfonic acid) (HEPES) buffer, then incubated with 5 μL of anti-CD62 P-FITC (Becton Dickinson & Company, New Jersey, United States of America) in the dark for 15 min Polyethyleneimine (PEI, 0.5 mg/mL) was utilized to activate the PRP as a positive control, and anti-IgG-FITC was used as a nonspecific binding control.

### 2.7 Activation of complement by PDA NPs

The PPP obtained by centrifugation was mixed with equal volume of various concentrations of PDA NPs solutions. The mixture was incubated at 37°C for 30 min and then diluted (×1,000) with a dilution buffer from a commercial C3a enzyme immunoassay kit (eBioscience Inc., San Diego, United States of America). Next, 100 μL of the diluted samples were added to a well plate pre-coated with an anti-human C3a antibody and incubated for 1 h at 25°C. After incubation, the wells were washed five times. A biotin-conjugated detection antibody was added to the test wells, and incubated for 2 h at 25°C. The wells were then washed five times to remove any unbound antibody. Subsequently, streptavidin-horseradish peroxidase was added to the test wells, incubated for 2 h at 25°C, and then washed out. Finally, the substrate solution and stop buffer were sequentially added to the wells. The absorbance of each well at 450 nm was measured using a microplate reader. In this experiment, the inulin solution (10 mg/mL) and PBS treatment groups were used as positive and negative controls, respectively.

### 2.8 Acute toxicity analysis

Twelve C57BL/6 mice (20 ± 5 g) were randomly divided into four groups of three mice each. The mice in each group were injected via the tail vein with different doses of PDA NPs solution (0.6, 6, 30 mg/kg per mouse, approximately equal 0.01, 0.1, 1 mg/mL, respectively). Mice injected with PBS served as controls. Twenty-4 hours after injection, organs (heart, liver, spleen, lung, kidney) from each group were collected and fixed with 4% paraformaldehyde for H&E analysis.

### 2.9 Statistical analysis

The measurement data in this study were presented as mean ± standard deviation (SD). Statistical differences between groups were analyzed with SPSS 29.0 b y using one-way ANOVA (*p* < 0.05: *, *p* < 0.01: ** and *p* < 0.001: ***).

## 3 Results and discussion

### 3.1 Characterization of PDA NPs

**FIGURE 1:**
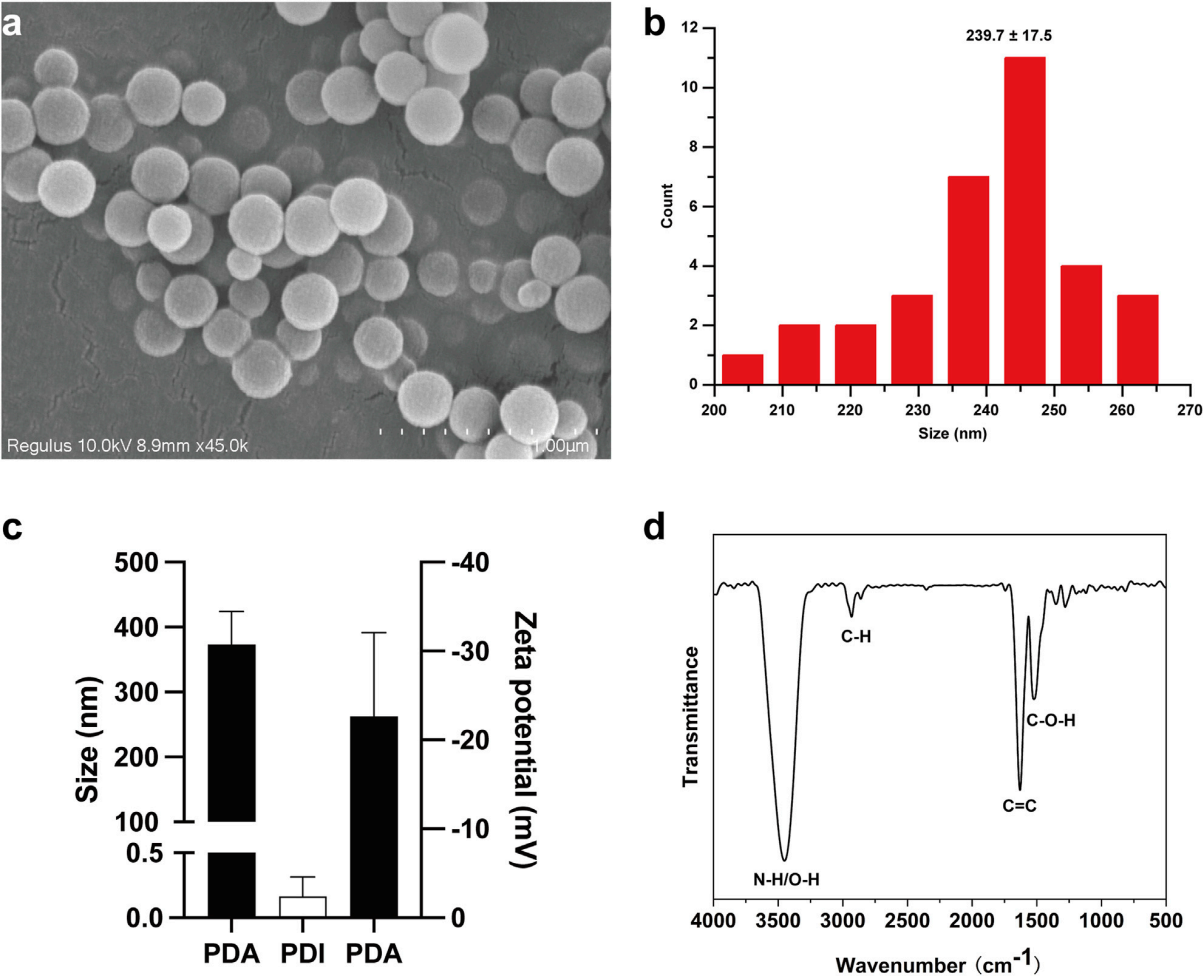
Synthesis of PDA NPs and their size. **(A)** SEM image of PDA NPs; **(B)** the corresponding diameter distribution histogram of PDA NPs, measured by ImageJ; **(C)** size and zeta-potential of PDA NPs detected by DLS; **(D)** FTIR spectra of PDA NPs.

Dopamine has been shown to polymerize into poly (dopamine) nanoparticles (PDA NPs) via oxidation-rearrangement-self-assembly processes in weakly alkaline aqueous solutions with air exposure (Mrówczyński, 2018). In this study, PDA NPs were rapidly synthesized through the oxidative self-polymerization of dopamine in an alkaline solution (pH 8.5). SEM was used to examine the size and morphology of the PDA NPs. SEM images and the corresponding diameter distribution histogram, obtained using ImageJ, are presented in Figures 1A, B, respectively. The data revealed that the PDA NPs were monodisperse, uniform, and spherical, with an average diameter of 239.7 ± 17.5 nm. DLS measurements of the size and zeta potential of PDA NPs in solution showed sizes of 373 ± 41 nm (PDI: 0.16 ± 0.12) and a zeta potential of −22 ± 7.7 mV, as illustrated in Figure 1C. FTIR was employed to characterize the chemical structure and surface functional groups of the PDA NPs. The FTIR spectrum in Figure 1D shows strong absorption peaks at 3,500–3,000 cm^-1^, corresponding to the stretching vibrations of N-H and O-H bonds. Weaker peaks near 2,930 cm^-1^ and 2,859 cm^-1^ are attributed to C-H stretching vibrations. Strong absorptions near 1,626 cm^-1^ and 1,516 cm^-1^ are associated with 5,6-dihydroxyindole, intermediates of oxidative self-polymerization. Peaks within the range of 1,610–1,000 cm^-1^ reflect hydrogen bonding interactions. These results indicate that PDA NPs have numerous surface functional groups, such as hydroxyl and amino groups, enabling further reactions like amidation, esterification, and Michael addition under appropriate conditions (Liu et al., 2016).

### 3.2 Impact of PDA NPs on RBC morphology and hemolysis

RBCs are crucial for oxygen and carbon dioxide transport in vertebrates and also have immune functions. The biconcave shape of human RBCs is essential for efficient gas exchange and oxygen uptake (Li et al., 2015). This study assessed the effects of PDA NPs on RBC morphology and membrane structure by examining morphological changes and hemolysis rates.

#### 3.2.1 Morphological changes

**FIGURE 2:**
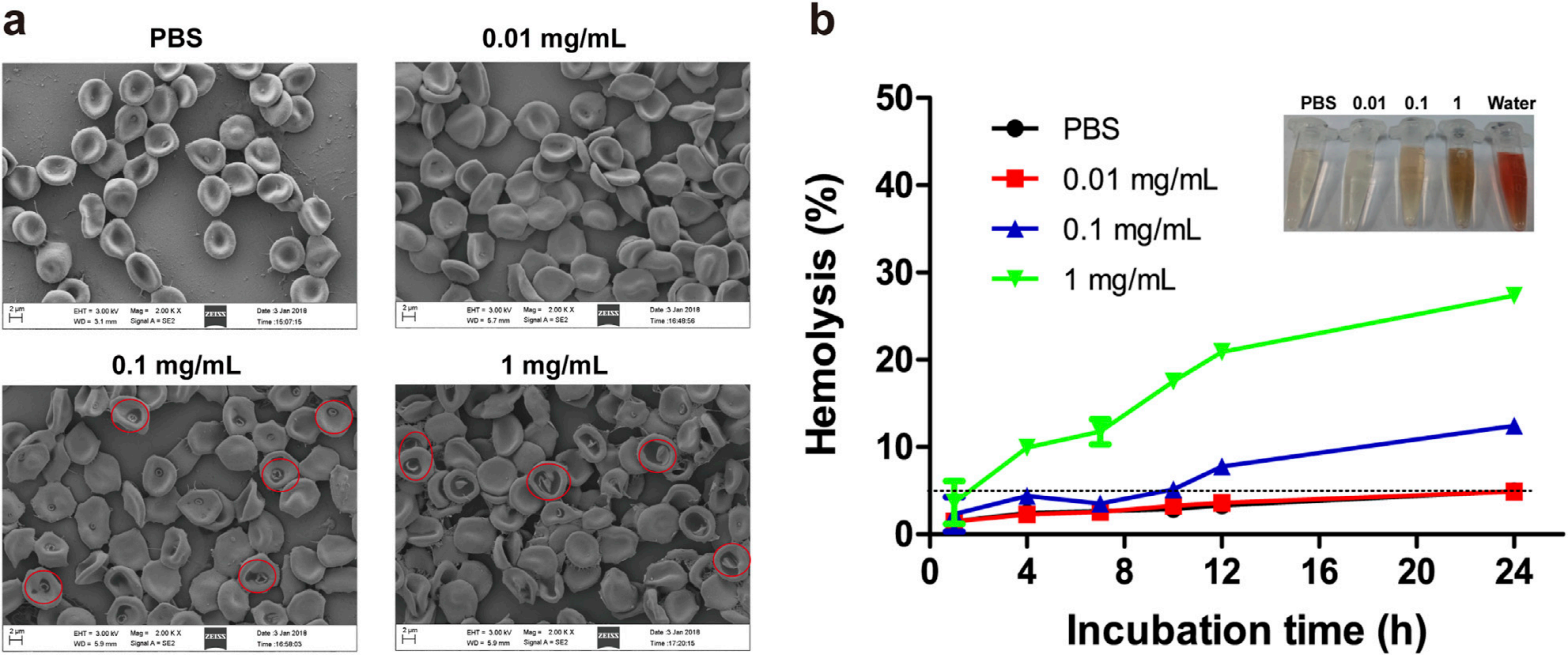
Effect of PDA NPs on RBC morphology and lysis. **(A)** Morphology and aggregation of the RBCs in the presence of different concentrations of PDA NPs as observed with SEM. **(B)** Hemolysis of the RBCs incubated with different concentrations of PDA NPs at room temperature.

The effects of varying concentrations of PDA NPs on RBC morphology were investigated. As shown in Figure 2A, RBCs incubated with 0.01 mg/mL of PDA NPs exhibited no significant morphological changes compared to the control group. At 0.1 mg/mL, RBCs displayed edge spikes, shrinkage, and surface blistering. At 1 mg/mL, RBCs showed severe shrinkage, surface blistering, dehydration, and adhesion. These observations suggest that PDA NPs interact strongly with the RBC membrane, affecting their morphology in a concentration-dependent manner.

#### 3.2.2 Hemolysis rate

When nanomaterials enter the body and contact RBCs, they can cause the rupture and lysis of RBC membranes, leading to the release of hemoglobin. The RBC hemolysis assay is an intuitive and convenient method commonly utilized to assess the degree of damage to cell membranes caused by nanomaterials (Sæbø et al., 2023). In this study, we further investigated the hemolysis rate induced by different concentrations of PDA NPs co-incubated with RBCs for various durations. As shown in Figure 2B, the hemolysis rate of RBCs in PBS or 0.01 mg/mL PDA NPs was less than 5% even after 24 h of incubation. In contrast, RBCs exposed to 0.1 mg/mL PDA NPs showed hemolysis at 12 h, with the hemolysis rate reaching 12.45% at 24 h. Furthermore, RBCs in 1 mg/mL PDA NPs began to lyse rapidly and significantly, with the hemolysis rate reaching 27.35% at 24 h. These results demonstrate that RBC lysis in the presence of PDA NPs was both concentration- and time-dependent.

It has been reported that the interaction between biomaterials and RBCs can be mediated by electrostatic attraction, hydrophobic interactions, H-bonds and van der Waals forces (Liu et al., 2010). Combining the results of RBC morphology and hemolysis, it was found that PDA NPs can destroy RBC membrane structure and integrity in a concentration- and time-dependent manner. The effect may be related to the abundant active groups such as catechol, hydroxyl, imino, and amino groups on the surface of PDA NPs (Cheng et al., 2019), which facilitate their adsorption to the RBC membrane and interaction with macromolecules like proteins, thereby disrupting and altering the components of RBC membrane. Additionally, the molecular structure of PDA NPs includes numerous benzene ring structures with strong hydrophobicity (Cheng et al., 2019), which can interact with the lipid layer of RBCs through hydrophobic interactions, leading to changes in RBC morphology or lysis of the RBC membrane.

### 3.3 Effect of PDA NPs on fibrinogen structure and conformation

After entering the bloodstream, biomaterials will inevitably contact and/or interact with proteins present in the blood. Fibrinogen, a glycoprotein, is a crucial component involved in coagulation and hemostasis. It is converted by activated thrombin to water-insoluble fibrin, which forms a three-dimensional clot with activated platelets (Davie and Fujikawa, 1975). The physiological function of a protein is generally determined by its structure, making the structure and conformation of fibrinogen critical to its function. Therefore, it is essential to study the impact of biomaterials on fibrinogen’s structure and conformation to understand how biomaterials affect the body’s coagulation function.

#### 3.3.1 UV-vis absorption spectroscopy

**FIGURE 3:**
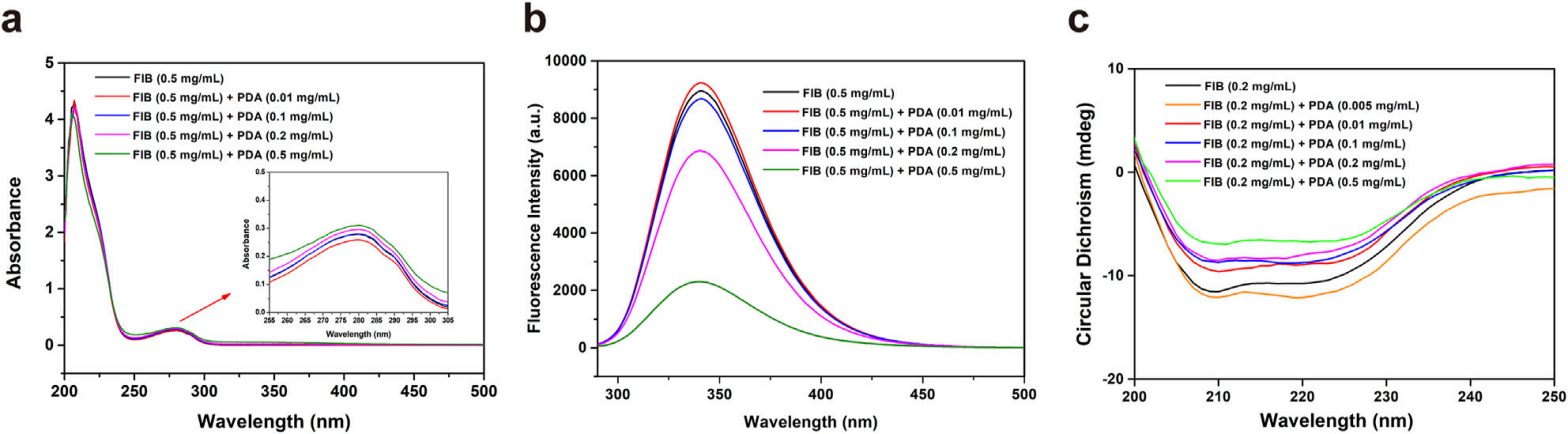
UV-vis spectra **(A)**, fluorescence spectra **(B)** and CD spectra **(C)** of fibrinogen (FIB) in the absence and presence of PDA NPs.

UV-vis absorption spectroscopy is a method for analyzing the composition of substances based on their varying degrees of absorption of different wavelengths of UV-vis. Figure 3A showed the effect of different concentrations of PDA NPs on the UV-vis absorption of fibrinogen. As the concentration of PDA NPs increased, the absorption intensity of fibrinogen at 280 nm gradually increased, while the peak position remained unchanged. The absorption peak of fibrinogen at 280 nm is primarily due to transitions of tryptophan and tyrosine residues in its peptide chain. The absorption intensity at 280 nm is related to the polarity of the microenvironment surrounding the benzene heterocycles of these aromatic amino acids (Cai and Zhang, 2017). Therefore, these results demonstrate that the interaction of PDA NPs with fibrinogen can change the microenvironment of tryptophan and tyrosine in a concentration-dependent manner.

#### 3.3.2 Fluorescence spectroscopy

Fluorescence spectroscopy is a common method for examining protein conformational changes, reflecting the distribution of fluorescence intensity of fluorescent molecule under a fixed excitation wavelength. When fluorescent molecules are irreversibly damaged by internal factors and/or external factors, fluorescence quenching occurs, resulting in a decrease in fluorescence intensity. Figure 3B showed the fluorescence emission spectra of fibrinogen in the absence and presence of PDA NPs. The fluorescence intensity at 340 nm decreased with increasing concentration of PDA NPs, while the fluorescence emission peak position remains unchanged. Tryptophan residues, located in the hydrophobic core of fibrinogen, are the main fluorescent groups excited at 280 nm and are extremely sensitive to the surrounding microenvironment (Khrustalev et al., 2019). The results suggest that PDA NPs can alter the local microenvironment of tryptophan residues, causing them to become exposed from the hydrophobic core to the surrounding polar solvent.

#### 3.3.3 CD spectroscopy

A CD spectrometer is an effective tool for detecting the effect of biomaterials on protein structure and conformation at the molecular level. The CD spectrum of pure fibrinogen exhibits two distinct negative peaks at 208 nm and 222 nm, characteristic of its α-helix structure. To study the influence of PDA NPs on the secondary structure of fibrinogen, we obtained CD spectra for pure fibrinogen and fibrinogen/PDA NPs mixtures (Figure 3C). As the concentration of PDA NPs increased, the characteristic negative peaks of the fibrinogen α-helix structure at 208 nm and 222 nm diminished in ellipticity, suggesting a dose-dependent interaction. This result indicates that while PDA NPs can induce changes in the local environment and potentially the tertiary structure of fibrinogen, they have a more pronounced effect on the protein’s α-helical content. The preservation of the peak positions during the CD analysis indicates that the overall fold of the protein may not be drastically altered, but the reduction in ellipticity suggests a loss of ordered α-helical structure, which could impact fibrinogen’s function in the coagulation cascade.

The results highlight the intricate relationship between the chemistry of PDA NPs and their interactions with fibrinogen. The surface groups of PDA NPs, such as catechols, hydroxyl, and amino groups, can interact with the protein’s aromatic residues (e.g., tryptophan and tyrosine), altering its local environment. These interactions could lead to conformational changes in fibrinogen, affecting its secondary structure and potentially impairing its function in coagulation. The concentration-dependent effects observed across UV-vis, fluorescence, and CD spectroscopy suggest that the interaction between PDA NPs and fibrinogen is not a simple adsorption process but involves complex changes in the protein’s structure.

### 3.4 Effect of PDA NPs on blood coagulation

#### 3.4.1 Blood plasma coagulation

**FIGURE 4:**
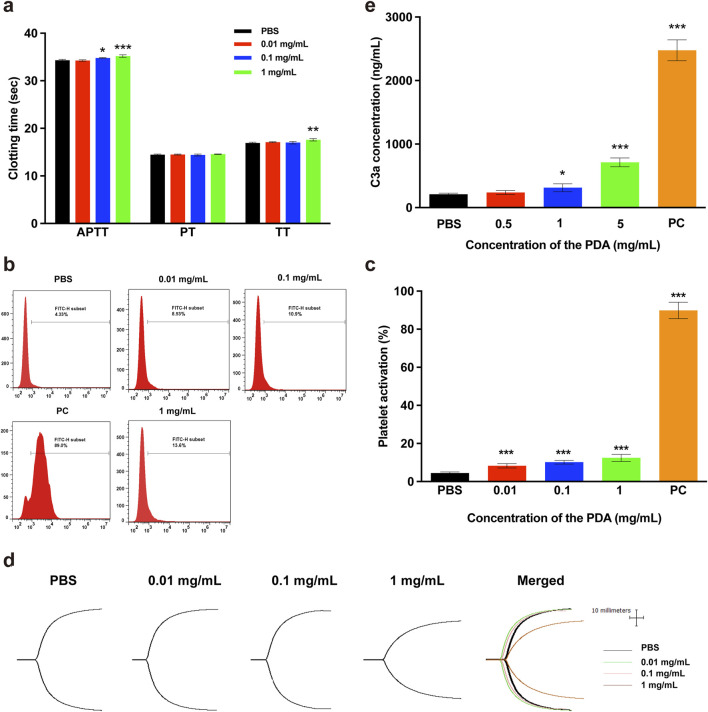
Effect of PDA NPs on blood coagulation and complement system. **(A)** APTT, PT and TT of plasma coagulation in the presence of PDA NPs with PBS as a negative control. **(B, C)** Platelet activation by PDA NPs with PBS as a negative control and PEI as a positive control. **(D)** TEG traces of whole blood coagulation in the presence of PDA NPs with PBS as a control. **(E)** Complement activation by PDA NPs with PBS as a negative control and inulin as a positive control.

The APTT, PT and TT are critical indicators for clinically monitoring blood coagulation function. APTT and PT values represent the level of the extrinsic or intrinsic coagulation pathway of blood plasma, respectively (Zhong et al., 2013). TT values indicate the ability of plasma fibrinogen to convert to fibrin. To evaluate the effects of PDA NPs on blood plasma coagulation, we measured the blood plasma coagulation function indicators APTT, PT and TT were measured in this study. As shown in Figure 4A, when the concentration of PDA NPs was 0.1 mg/mL or higher, the clotting time for the APTT indicator significantly increased compared to the control group. Additionally, PDA NPs caused a significant increase in the clotting time of TT indicator when their concentration reached 1 mg/mL. However, PDA NPs did not have a significant effect on PT indicators within the tested concentration range. The results demonstrate that PDA NPs exhibit a concentration-dependent anticoagulant effect. This may be due to the interaction of PDA NPs with fibrinogen and/or clotting factors upon exposure to blood plasma, impairing their normal biological functions.

#### 3.4.2 Platelet activation

The primary function of platelets is to promote hemostasis and accelerate coagulation. Typically, platelets are round, but upon contact with a wound surface or non-vascular intimal surface, they quickly aggregate, extend multiple pseudopods, and transform into dendritic platelets, with many particles expressed or released immediately. Among these particles, P-selectin (CD62 P), a glycoprotein on platelet α-granular membranes, is rapidly expressed on the plasma membrane and mediates platelet adhesion after stimulation by the external environment. In this study, the expression of the membrane protein CD62 P was measured to evaluate the degree of platelet activation by different concentrations of PDA NPs. The representative flow cytometry peaks and histogram of platelet activation rates were showed in Figures 4B, C, respectively. As shown in Figure 4C, the PDA NPs-treated groups exhibited significant platelet activation even at concentrations as low as 0.01 mg/mL compared to the negative control group. When the concentration of PDA NPs was 0.01 mg/mL or 1 mg/mL, the platelet activation ratios were near 2-fold (8.53%) or 3-fold (13.6%) that of the PBS treatment group (4.33%), respectively. However, the platelet activation ratios in the PDA NPs-treated groups were significantly lower than that of the positive control group (89.0%). The results indicate that platelets were sensitive to and easily activated by PDA NPs, suggesting an interaction between the platelets and PDA NPs, although this interaction was not as strong as the interaction between the platelets and cationic PEI.

#### 3.4.3 TEG

The TEG is a rapid, accurate, and sensitive assay utilized to monitor the coagulation function of whole blood by examining dynamic processes such as platelet aggregation, coagulation, and fibrinolysis. The results of a TEG assay include four key parameters: R, which refers to the time required from the addition of calcium chloride to the formation of the initial fibrin; K, indicating the time taken for the clot to reach a fixed strength; the α angle, which indicates the rate at which fibrin cross-links into clots; and MA, representing the maximum strength of the blood clot (Li et al., 2015). TEG dynamic coagulation traces and the corresponding R, K, α angle and MA values of whole blood after exposure to different concentrations of PDA NPs were showed in Figure 4D and Table 1, respectively. The results indicated that, compared to the normal reference values, there were no obvious abnormalities when the concentration of PDA NPs was less than 1 mg/mL. However, at a concentration of mg/mL, the K value was higher (indicating lower fibrinogen activity) and the MA value was lower (indicating lower platelet activity) than the normal range. From the results, we can infer that when the concentration of PDA NPs was less than 1 mg/mL, there is no significant effect on the coagulation function of whole blood.

**TABLE 1:** Clotting kinetics values of human whole blood containing PDA NPs.

Samples	R (min)	K (min)	α (deg)	MA (mm)
normal range	5–10	1–3	53–72	50–70
PBS control	7.1	1.7	66.3	63.7
0.01 mg/mL PDA NPs	5.3	1.4	69.6	63.4
0.1 mg/mL PDA NPs	5.8	1.6	68.2	62.0
1 mg/mL PDA NPs	7.5	3.1 ↑	56.2	48.7 ↓

The sign ↓ indicates a low value and ↑ a high value compared with the normal range provided by the TEG, analyzer.

This study explored the effects of various concentrations of PDA NPs on coagulation-related indices after contact with plasma, platelets, and whole blood *in vitro*. The results demonstrated that PDA NPs had a concentration-dependent effect on blood coagulation. At a low concentration (0.1 mg/mL), PDA NPs significantly affected coagulation factors, proteins and platelets. However, when PDA NPs contacted whole blood, a higher concentration (1 mg/mL) was required to show an effect on coagulation function. The observed coagulant and platelet activation effects of PDA NPs can be attributed to the nanoparticles' surface chemistry and material structure. The functional groups on the surface of PDA NPs, including catechols, hydroxyl groups, and amino groups, are known to engage in hydrogen bonding, electrostatic interactions, and hydrophobic interactions with blood proteins, coagulation factors, and platelets. These interactions can alter the normal function of these components, leading to changes in clotting time, platelet activation, and clot strength. This study provided reference information for the potential effects of PDA NPs on the blood coagulation system and function after entering the human body, and offers a basis for guiding the safe use of PDA NPs *in vivo*.

### 3.5 Activation effect of PDA NPs on complement system

The complement system is a protein response network consisting of over 30 proteins found in serum, tissue fluid, and on cell membranes surfaces, with a precise regulatory mechanism. This system plays a crucial role in inflammatory response, immune regulation, and blood coagulation, serving as an important effector and amplification system within the body. The activation of the complement system involves three pathways: the classical pathway, the alternative pathway, and the lectin pathway. Complement component C3 is the most abundant protein in the system and is cleaved by C3 convertase into C3a, which remains stable in plasma, and C3b, which is not stable in plasma. These components are involved in all three activation pathways. In biosafety evaluations of various biomaterials, the concentration of protein C3a is often measured to indicate the degree of complement system activation by these biomaterials. In this study, complement activation by PDA NPs was assessed by measuring the C3a concentration, with results shown in Figure 4E. The findings demonstrated that C3a concentration in blood plasma exposed to 1 and 5 mg/mL of PDA NPs were significantly higher than those in the negative control. In contrast, C3a concentrations in plasma exposed to 0.5 mg/mL of PDA NPs showed no significant difference compared to the negative control. This indicates that 1 and 5 mg/mL of PDA NPs significantly activated the complement system. It has been reported that the hydrophobicity of nanomaterial surfaces and the presence of surface functional groups, such as amino, carboxyl, and hydroxyl groups, are key determinants of their ability to activate the complement system (Toda et al., 2008; Moghimi et al., 2011). The activation effect of PDA NPs on the complement system is likely related to their surface’s abundant reactive groups, including catechol, hydroxyl, and amino groups, as well as their hydrophobic structure.

### 3.6 H&E analysis

**FIGURE 5:**
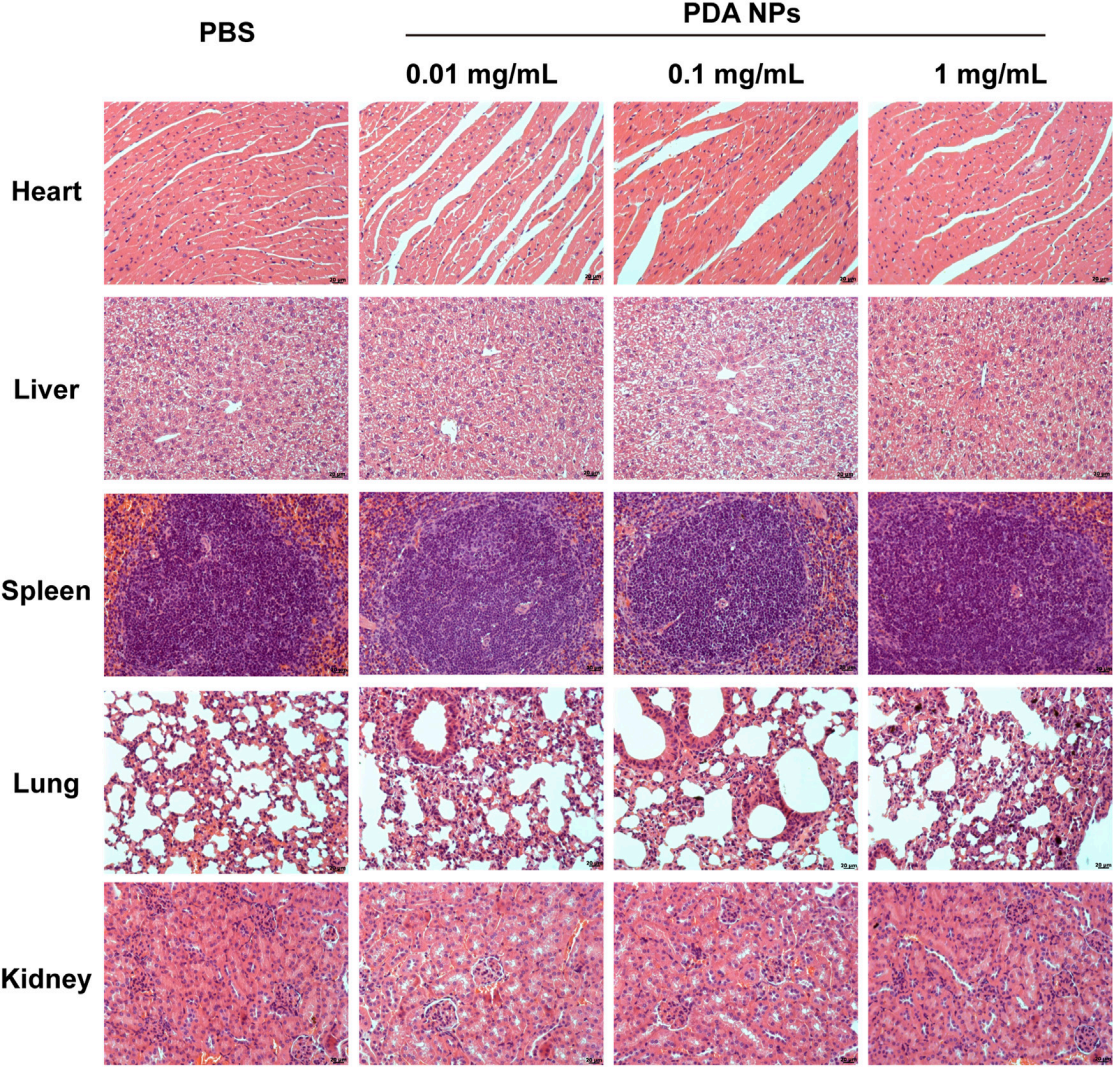
H&E analysis of heart, liver, spleen, lung, and kidney obtained from mice injected with different concentrations of PDA NPs.

The histological examination (H&E analysis) was performed on organs (heart, liver, spleen, lung, kidney) collected from C57BL/6 mice 24 h post-injection of PDA NPs at varying doses. The analysis aimed to assess potential acute toxicological effects of PDA NPs on the organs. As shown in Figure 5, the results showed no significant pathological changes or tissue damage in the organs of mice treated with PDA NPs (0.01, 0.1, 1 mg/mL, respectively) compared to the control group. This suggests that PDA NPs do not induce significant organ acute toxicity at the tested doses, indicating their potential safety for *in vivo* applications.

## 4 Conclusion

The findings from this study contribute to a better understanding of the interaction between PDA NPs and blood components. The concentration-dependent effects observed in the study highlight the importance of dose optimization for the safe use of PDA NPs in medical applications. The minimal impact on fibrinogen’s secondary structure α-helix suggests that PDA NPs may not significantly interfere with the protein’s physiological function. However, the significant activation of the complement system at higher concentrations of PDA NPs indicates a potential for immune response, which needs to be considered in the design of PDA NP-based therapies. The lack of acute toxicity observed in the H&E analysis supports the biocompatibility of PDA NPs but further long-term studies are warranted to fully understand their safety profile.

In conclusion, this study provides a comprehensive evaluation of the blood compatibility of PDA NPs, a critical aspect for their application in biomedical fields. The results demonstrate that PDA NPs have the potential for safe use in various medical applications, particularly at lower concentrations. However, caution must be exercised at higher concentrations due to the observed effects on blood coagulation and complement system activation. Further research is needed to explore the long-term effects and optimize the use of PDA NPs in clinical settings.

## Data Availability

The raw data supporting the conclusions of this article will be made available by the authors, without undue reservation.
